# Supplementary material for: Antibiotic Susceptibility Profiles and Resistance Mechanisms to β-Lactams and Polymyxins of Escherichia coli from Broilers Raised under Intensive and Extensive Production Systems
Source: Microorganisms. 2022 Oct 16;10(10):2044. doi: 10.3390/microorganisms10102044 (PMC9608943; doi:10.3390/microorganisms10102044)
Supplement: Supplementary file 1 [file microorganisms-10-02044-s001.zip › microorganisms-1937852-supplementary.pdf]

Table S1. Primers used for DNA amplification

Gene	Primer	Sequence (5'- 3')	[Primer]	Product (bp)	Annealing Temp.	References
CTX-M-1	MultiCTXMGP1_F<br>MultiCTXMGP1-2_R	TTAGGAARTGTGCCGCTGYA<br>CGATATCGTTGGTGGTRCCAT	0.4µM	688	60°C	Dallenne et al, 2010
CTX-M-2	MultiCTXMGP2_F<br>MultiCTXMGP1-2_R	CGTTAACGGCACGATGAC<br>CGATATCGTTGGTGGTRCCAT	0.2 µM<br>0.4 µM	404	60°C	Dallenne et al, 2010
CTX-M-9	MultiCTXMGP_F<br>MultiCTXMGP_R	TCAAGCCTGCCGATCTGGT<br>TGATTCTCGCCGCTGAAG	0.4 µM	561	60°C	Dallenne et al, 2010
CTX-M-8/25	CTX-Mg8/25_F<br>CTX-Mg8/25_R	AACRCRCAGACGCTCTAC<br>TCGAGCCGGAASGTGTYAT	0.4 µM	326	60°C	Dallenne et al, 2010
TEM	Primer TEM_F<br>Primer TEM_R	CATTTCCGTGTCGCCCTTATTC<br>CGTTCATCCATAGTTGCCTGAC	0.4 µM	800	60°C	Dallenne et al, 2010
SHV	Primer SHV_F<br>Primer SHV_R	AGCCGCTTGAGCAAATTAAAC<br>ATCCCGCAGATAAATCACCAC	0.4 µM	713	60°C	Dallenne et al, 2010
OXA	Primer OXA_F<br>Primer OXA_R	GGCACCAGATTCAACTTTCAAG<br>GACCCCAAGTTTCCTGTAAGTG	0.4 µM	564	60°C	Dallenne et al, 2010
ACC	MultiCaseACC_F<br>MultiCaseACC_R	CACCTCCAGCGACTTGTTAC<br>GTTAGCCAGCATCACGATCC	0.2 µM	346	60°C	Dallenne et al, 2010
FOX	MultiCaseFOX_F<br>MultiCaseFOX_R	CTACAGTGCGGGTGGTTT<br>CTATTTGCGGCCAGGTGA	0.5 µM	162	60°C	Dallenne et al, 2010
MOX; CMY-1, -8, -11; -19	MultiCaseMOX_F<br>MultiCaseMOX_R	GCAACAACGACAATCCATCCT<br>GGGATAGGCGTAACTCTCCCAA	0.2 µM	895	60°C	Dallenne et al, 2010
DHA	MultiCaseDHA_F<br>MultiCaseDHA_R	TGATGGCACAGCAGGATATTC<br>GCTTTGACTCTTTCGGTATTCG	0.5 µM	997	60°C	Dallenne et al, 2010
LAT-1; -3, BIL-1, CMY-2 to -7, -12 to -18, -21 to -23	MultiCaseCIT_F<br>MultiCaseCIT_R	CGAAGAGGCAATGACCAGAC<br>ACGGACAGGGTTAGGATAGY	0.2 µM	538	60°C	Dallenne et al, 2010
ACT-1; MIR-1	MultiCaseEBC_F<br>MultiCaseEBC_R	CGGTAAAGCCGATGTTGCG<br>AGCCTAACCCCTGATACA	0.2 µM	683	60°C	Dallenne et al, 2010

MCR-1	MCR1_F<br>MCR1_R	AGTCCGTTTGTTCTTGTGGC<br>AGATCCTTGGTCTCGGCTTG	0.5 µM	320	58°C	Rebelo et al, 2018
MCR-2	MCR2_F<br>MCR2_R	CAAGTGTGTTGGTCGCAGTT<br>TCTAGCCCGACAAGCATACC	0.5 µM	715	58°C	Rebelo et al, 2019
MCR-3	MCR3_F<br>MCR3_R	AAATAAAAATTGTTCCGCTTATG<br>AATGGAGATCCCCGTTTTT	0.5 µM	929	58°C	Rebelo et al, 2020
MCR-4	MCR4_F<br>MCR4_R	TCACTTTCATCACTGCGTTG<br>TTGGTCCATGACTACCAATG	0.5 µM	1116	58°C	Rebelo et al, 2021
MCR-5	MCR5_F<br>MCR5_R	ATGCGGTTGTCTGCATTTATC<br>TCATTGTGGTTGTCCTTTTCTG	0.5 µM	1644	58°C	Lu X et al, 2017
Int1	IntI1_F<br>IntI1_R	GGTCAAGGATCTGGATTTCG<br>ACATGCGTGTAAATCATCGTC	0.4 µM	436	60°C	Kargar et al, 2014
Int2	IntI2_F<br>IntI2_R	CACGGATATGCGACAAAAAGG<br>TGTAGCAAACGAGTGACGAAATG	0.4 µM	788	60°C	Kargar et al, 2014
Int3	IntI3_F<br>IntI3_R	AGTGGGTGGCGAATGAGTG<br>AGTGGGTGGCGAATGAGTG	0.4 µM	600	60°C	Kargar et al, 2014

Table S2. Primers for Sanger sequencing.

Gene	Primer	Sequence	[Primer]	Product (bp)	Annealing Temp.	Reference
TEM	FIN<br>DEB	ATTCTTGAAGACGAAAGGGC<br>ATGAGTAAACTTGGTCTGAC	0.4 µM	1091	54°C	Caniça et al, 1997
SHV	SHVF149P<br>SHVR1059	CGCTTCTTTACTCGCCTTTA<br>TTAGCGTTGCCAGTGCTC	0.5 µM	911	56°C	Rasheed et al, 1997
CMY	CMYG2F<br>CMYG2R	TTACGGAACTGATTTCATG<br>TCGTCAGTTATTGCAGC	0.5 µM	1143	56°C	Manageiro, 2011
AmpC	Int-B2<br>Int-HN	TTCCTGATGATCGTTCTGCC<br>AAAAGCGGAGAAAAGGTCCG	0.5 µM	1315	57°C	Mammeri et al, 2006
CTX-M-1	CTXM15F<br>CTXM1R	AGAATAAGGAATCCCATGGTT<br>CCGTTTCCGCTATTACAA	0.5 µM	903	53ºC	Mendonça et al, 2007
CTX-M-9	CTXM9F<br>CTXMG9R2	ATGGTGACAAAGAGAGTGCAAC<br>AGTTACAGCCCTTCGGCGAT	0.5 µM	878	55°C	Cottel et al, 2012
CTX-M-8/25	CTXM8/25F<br>CTXM8/25R	GATRYTAATGACRACRGCCT<br>TGGGTGAAGTAAGTSACCAG	0.5 µM	777	57°C	This study

[a] **Y**=T or C; **R**=A or G; **S**=G or C; **D**=A or G or T
F, forward; **R**, reverse

Table S3. Prevalence of multidrug resistance patterns observed in *E. coli* isolates from broilers raised under intensive (n=77) and extensive production systems (n=42).

Multidrug resistance patterns	Extensive system		Intensive system		Total	
	N	%	N	%	N	%
AMP-CIP-NAL-TET	7	16.6	2	2.6	9	7.6
AMP-CIP-SMX-TMP	1	2.4	0	0	1	0.8
AMP-CIP-NAL-SMX	1	2.4	1	1.3	2	1.7
AMP-SMX-TET-TMP	2	4.8	2	2.6	4	3.4
AMP-CIP-GEN-NAL	1	2.4	0	0	1	0.8
AMP-CHL-CIP-NAL	0	0	1	1.3	1	0.8
CIP-GEN-NAL-SMX	1	2.4	0	0	1	0.8
CIP-NAL-SMX-TET	2	4.8	1	1.3	3	2.5
GEN-SMX-TET-TMP	0	0	1	1.3	1	0.8
AMP-CIP-NAL-SMX-TET	2	4.8	1	1.3	3	2.5
AMP-CIP-NAL-SMX-TMP	1	2.4	1	1.3	2	1.7
AMP-CHL-CIP-NAL-SMX	0	0	2	2.6	2	1.7
AMP-CIP-NAL TET-TMP	1	2.4	3	3.9	4	3.4
AMP-CIP-SMX-TET-TMP	0	0	1	1.3	1	0.8
AMP-CHL-CIP-NAL-TET	0	0	1	1.3	1	0.8
AMP-AZI-CIP-NAL-TMP	0	0	1	1.3	1	0.8
AMP-CIP-NAL-SMX-TET-TMP	8	19	7	9.1	15	12.6
AMP-CHL-CIP-NAL-SMX-TMP	1	2.4	0	0	1	0.8
AMP-CHL-CIP-GEN-NAL-SMX	0	0	1	1.3	1	0.8
AMP-CHL-CIP-NAL-SMX-TET	0	0	1	1.3	1	0.8
AMP-AZI-CHL-NAL-SMX-TMP	0	0	1	1.3	1	0.8
AMP-AZI-CIP-NAL-SMX-TET-TMP	0	0	1	1.3	1	0.8
AMP-CHL-CIP-NAL-SMX-TET-TMP	2	4.8	9	11.7	11	9.2
AMP-AZI-CIP-GEN-NAL-SMX-TET-TMP	1	2.4	4	5.2	5	4.2
AMP-FOT-TAZ-CIP-SMX-FEP	0	0	1	1.3	1	0.8
AMP-FOT-TAZ-CHL-GEN-SMX-FEP	0	0	1	1.3	1	0.8
AMP-FOT-TAZ-CHL-CIP-NAL-SMX-FEP	0	0	1	1.3	1	0.8
AMP-FOT-TAZ-CHL-CIP-SMX-TET-FEP	0	0	2	2.6	2	1.7
AMP-FOT-TAZ-CIP-SMX-TMP-FEP-FOX	0	0	1	1.3	1	0.8
AMP-FOT-TAZ-CHL-SMX-TET-FEP-FOX	0	0	1	1.3	1	0.8
AMP-FOT-TAZ-CIP-NAL-SMX-TMP-FEP	1	2.4	1	1.3	2	1.7

AMP-FOT-TAZ-CIP-NAL-SMX-TET-FEP	2	4.8	2	2.6	4	3.4
AMP-FOT-TAZ-CIP-NAL-SMX-TET-FEP-FOX	0	0	1	1.3	1	0.8
AMP-FOT-TAZ-CIP-NAL-SMX-TMP-FEP-FOX	0	0	1	1.3	1	0.8
AMP-FOT-TAZ-CIP-NAL-SMX-TET-TMP-FEP	3	7.1	1	1.3	4	3.4
AMP-FOT-TAZ-CHL-CIP-NAL-SMX-TET-FEP	1	2.4	10	13	11	9.2
AMP-AZI-FOT-TAZ-CHL-CIP-SMX-TET-TMP-FEP	0	0	1	1.3	1	0.8
AMP-FOT-TAZ-CHL-CIP-NAL-SMX-TET-FEP-FOX	0	0	1	1.3	1	0.8
AMP-FOT-TAZ-CIP-NAL-SMX-TMP-FEP-FOX-ETP	0	0	1	1.3	1	0.8
AMP-FOT-TAZ-CHL-CIP-NAL-SMX-TET-TMP-FEP	0	0	2	2.6	2	1.7
AMP-FOT-TAZ-CHL-CIP-COL-NAL-SMX-TET-FEP	0	0	1	1.3	1	0.8
AMP-AZI-FOT-TAZ-CIP-NAL-SMX-TET-TMP-FEP	1	2.4	0	0	1	0.8
AMP-AZI-FOT-TAZ-CHL-CIP-NAL-SMX-TET-TMP-FEP	2	4.8	0	0	2	1.7
AMP-AZI-FOT-TAZ-CHL-CIP-GEN-NAL-SMX-TET-TMP-FEP	1	2.4	5	6.5	6	5
AMP-FOT-TAZ-CHL-CIP-GEN-NAL-SMX-TET-TMP-FEP-FOX	0	0	1	1.3	1	0.8

AMP, Ampicilin; AZI, Azithromycin; FOT, Cefotaxime; TAZ, Ceftazidime; CHL, Chloramphenicol; CIP, Ciprofloxacin; GEN, Gentamicin; NAL, Nalidixic Acid; SMX, Sulphamethoxazole; TET, Tetracycline; TMP, Trimethoprim; FEP, Cefepime; FOX, Cefoxitin
